# Editorial

**Published:** 1876-06

**Authors:** 


### Editorial.

### MICHIGAN UNIVERSITY MEDICAL COLLEGE.

This Institution is undergoing an ordeal from which we hope it will pass unscathed and unharmed.

It is now receiving the open opposition, hostility and abuse from a certain class of medical men throughout the country; many of whom, doubtless, have been the covert enemies of the College, who are now greatly gratified that an occasion has occurred, upon which they might seize as an excuse for open hostility, even though it might result in the destruction of the Institution.

This excuse is found in the establishment of two chairs for teaching Homœopathy, under the care and direction of the University. This was accomplished by the passage of a law to that effect by the Legislature of Michigan, and an appropriation of $6,000 per year for sustaining the enterprise. The demands upon the regents to carry out the provisions of this law were loud and urgent, and they acted in good faith, in the least objectionable way apparent to them; but all the while against the earnest protest of the regular Medical Faculty, or at least all of them who were cognizant of what was being done. And now, the establishment being accomplished, what are the facts as developed during the last six months, or from October last to March 31st ensuing. Simply this, that two homœopathic teachers appeared, took their places, and proceeded to teach, according to their particular system, to about twenty students. They had no official connection, nor any other for that matter, with the regular Faculty. The foundation branches of medicine—anatomy, physiology, pathology, surgery, obstetrics and chemistry, were not taught by the homœopathic professors, but *all* the medical and dental students received their instruction in these branches from the regular medical Faculty.

This is the extent of the transgression of the Faculty of the Medical College of Michigan University, viz: That they consented to proceed with their regular work, in the presence of about twenty homœopathic students, when the fact is the Faculty have no control as to who shall enter, if they fulfill the requirements.

"But they ought to have resigned."

Why? Would it have been reasonable, just, or honorable for this body of teachers, equal in ability and reputation to any in this country, to have "stepped down and out," because forsooth, about twenty students who were studying Homœopathy, were present at their lectures? Was there any probability that these students would contaminate or demoralize any body? Their opportunities for this were certainly not very great. On the other hand, ten professors had a daily opportunity to correct any false principles that may have been imbibed, and properly educate these twenty seekers after truth and knowledge.

It would have been a most puerile course for the Faculty to have resigned and given up this grand and noble work, having a growth of nearly a third of a century, and becoming more and more efficient every year for the accomplishment of its appropriate work, simply because a few, or even a good many croakers and mal-contents saw fit to criticise and stir up strife and contention.

This alleged complicity with quackery is not the only, or even the chief ground of this opposition. There are several other things, that it has not been reckoned feasible to bring to the surface, that are operating with many to a far greater extent. The system, so nearly approximating free education, adopted by the State of Michigan, and applying to the Medical College of the University, as well as to other departments, has been the occasion of arousing somewhat of a hostile feeling on the part of Medical College interests throughout the country. This is a large and powreful influence.

That all this stir and hub-bub is purely and solely induced and stimulated by a jealous care and anxiety for the reputation, purity, honor and dignity of the medical profession, no intelligent and unprejudiced person will for one moment believe or admit.

It is a matter of regret that a few medical journals have taken up this controversy in such a partisan spirit and with such manifest prejudice, as to draw upon themselves the criticism, if nothing more, of every unbiased person, who may be familiar with the circumstances.

We trust and believe that the Medical College of Michigan, will stand unshaken by this storm, and be as popular and efficient for good in the future as hitherto.

### CALIFORNIA STATE DENTAL ASSOCIATION.

The seventh annual meeting of this body will be held in San Francisco, beginning on Tuesday, June 13th, at 10 o'clock and continue four days.

A most cordial invitation is extended to every worthy member of the profession to be present on that occasion. It is very desirable that all such be embraced in the membership of the society.

In order to become a member the applicant must be a graduate of a respectable dental college, or have been in practice three years, must have a good moral character, and be endorsed by two members of the association.

All the members must conform to the Code of Ethics of the American Dental Association.

The objects of the society, as stated in the constitution, are: "The promotion of the highest excellence in the science and art of dentistry and its collateral branches; the cultivation of close professional relation and good fellowship among our members, and collectively to represent and have cognizance of the common interests of the Dental profession."

These are objects certainly that should elicit the best efforts of earnest men.

We feel a warm interest in our profession in California, and in one sense, a double interest. It is always a matter of solicitude to see a profession grow and develop in a new country; and in addition to this, we have in that State, and especially in San Francisco, a large number of very warm friends, some of whom we have endeavored, in by-gone days, to assist in their preparation for professional work. All these we regard with an especial interest, and it is a matter of great gratification to know that nearly, if not quite all, of these are active workers in the State Association.

Long may this society live and prosper.

### INDIANA STATE DENTAL SOCIETY.

The eighteenth annual meeting of this Society will be held at Indianapolis, on Tuesday, June 27th, 1876, at 10 o'clock a. m., and continue its sessions during three days,

J. R. Clayton, Sec. J. K. Jameson, Pres.

ORDER OF BUSINESS.

1. Calling of the Roll.
2. Payment of Dues.
3. Reading of Minutes.
4. Reports of Standing Committees.
5. Reports of Special Committees.

Election of members at any time during the session, upon report of the Committee.

Members and others who have interesting cases in model, or drawing, or new instruments, or appliances, are requested to bring them.

SUBJECTS AND ESSAYISTS.

1. Operative Dentistry—S. B. Brown, S. T. Kirk and T. F. Hacker.
2. Dental Therapeutics—I. Knapp, W. E. Driscoll.
3. Microscopy—P. G. C. Hunt, J. R. Clayton, C. D. Watson.
4. Dental Education—W. C. Stanley, Merit Wells, J. A. Turner.
5. Mechanical Dentistry—M. H. Chappell, J. W. Keely, W. F. Morrell.

Other subjects will be discussed, and voluntary essays are solicited.

Arrangements have been made with the proprietors of the Remy Hotel, on Circle Street, to entertain all in attendance at $2.00 per day; regular price, $3.50.

All dentists not members of the society are most cordially invited to be present and participate in the discussions. The meeting will be one of unusual interest.

P. G. C. Hunt,
W. M. Herriott,
J. A. Turner,
Ex. Committee.

### NORTHERN OHIO DENTAL ASSOCIATION.

The seventeenth annual meeting of this Association will be held at the office of Dr. D. R. Jennings, 319 (new number) Euclid Avenue, Cleveland, Ohio, commencing on Tuesday June 13th, 1876, at 10 o'clock a. m. Session will continue two days, and a full attendance is desired. Members of the profession are cordially invited to be present.

ORDER OF BUSINESS.

Reading of minutes of last annual meeting.
Reports of Officers and Committees.
Reading of Essays and Discussions.
Miscellaneous Business.
Eelection of officers, 11 o'clock a. m. each day.

SUBJECTS FOR DISCUSSION.

Treatment of Exposed Dental Pulps.
Replanting and Transplanting Natural Teeth.
Methods and Appliances for Correcting Dental Irregularities.
The different Bases for Artificial Dentures.
Miscellaneous.

C. BUFFETT, Cor. Sec. E. J. WAYE, Pres.

### CORRESPONDENCE.

PORTSMOUTH, O., May 5, 1876.

EDITOR REGISTER:—I am informed that the agents for the Rubber Company are circulating the report that they had me before the U. S, Court, at Cincinnati, to show cause why I should not be attached for violating an injunction of the courts in using Celluloid, claiming that I had been enjoined for using the same. I wish to state, for the benefit of those concerned, that I have never been under injunction for using Celluloid, but am under injunction for using rubber, and was at Cincinnati for violating said order. But as will be seen by notice taken from Cincinnati *Times*, of the 4th inst., the rule was refused in my case. The following is the proceedings that are referred to, as reported to the Cincinnati *Times*, viz:—CONTEMPT.—Dr. W. W. Monroe, of Portsmouth, a dentist, recently sued by the Vulcanite Rubber Company for the infringement of its patent, was brought down from that place yesterday, and arraigned before Judge Swing on charge of contempt of Court. The testimony showed that there was no intention, on the part of the defendant, to disobey the order of Court, and Judge Swing consequently refused to grant the rule. W. W. MONROE.

### DENTAL ANNOUNCEMENT.

In accordance with an Act of the General Assembly, entitled, "An Act to regulate the practice of Dentistry, and protect the people against empiricism in relation thereto in the State of South Carolina," the South Carolina State Dental Association, and the State Board of Dental Examiners, will meet in the city of Greenville, at the dental rooms of Drs. Norwood & McDavid, at 8 p. m., on the second Tuesday in June, 1876.

The following committees will be expected to make a report on the subjects assigned:

COMMITTEE ON MEMBERSHIP.

Dr. J. W. Norwood—Caries.
" J. B. Patrick—Premature Loss of Teeth.
" W. S. Brown—Pivot Teeth, or the Mirror, (either,)
" J. S. Thompson—Our Correspondence.
" C. L. Boozer—Articulation.

COMMITTEE ON MECHANICAL DENTISTRY.

Dr. B. A. Muckenfuss—The Best Base.
" W. S. Reynolds—Selection of Artificial Teeth.
" E. C. Jones—Temporary Plates.
" J. H. Alexander—Objections to Artificial Teeth.
" S. M. Dinkins—Partial Sets.

COMMITTEE ON OPERATIVE DENTISTRY.

Dr. C. C, Patrick—Mechanical Appliances in Op. Dentistry.

Dr. J. R. Thompson—Conditions Demanding Contour Work; Cause of Failures.

Dr. H. B. Rice—Diagnosis and Treatment of Pulpititis and Periostitis.

Dr. T. Berwick Legare—Anæsthesia, or When to Extract.

Dr. D. R. McCallum—Removing Tartar.

EXECUTIVE COMMITTEE.

Dr. J. Q. McDavid—The Use of Phrenology to the Dentist.
" J. W. Crymes—Electricty.
" W. A. Williams—Impressions.
" H. D. Wilson—Diagnosis.
" H. B. Teague—Salicylic Acid.

To elevate the standard of a noble and humane profession is the object of this association.

This is the sixth year of our organization; we are a recognized corporate body, and must prove ourselves worthy of the confidence and trust reposed in us. Those who are members will attend if possible. Those who are not, we feel assured, will avail themselves of the earliest opportunity to join. You are earnestly invited to attend. Bring an account of your success that others may be encouraged; tell of your difficulties that you may be aided to overcome them.

G. F. S. Wright, D. D. S., President, Columbia.
J. W. Norwood, First Vice-President, Greenville.
B. H. Teague, D. D. S., Second Vice-President, Aiken.
J. S. Thompson; D. D. S., Corresponding Sec., Abbeville.
Theo. F, Chupein, D. D. S., Recording Sec., Charleston.
T. W. Boucher, Treasurer, Cheraw.

### POSTPONEMENT.

In consequence of the annual meeting of the New York and Pennsylvania State Dental Societies at the same time of meeting of the New Jersey State Dental Society, the latter has been postponed one week, July 18th, 1876.

Chas. A. Meeker, Sec.

### PERSONAL.

By a private note we learn that Dr. W. C. Horne, D. D. S., formerly of New York, has established himself in business in Rome, Italy.

The Doctor was one of the most active and efficient members of the dental profession in this country; and will doubtless accomplish his purpose in his new field of labor. He is a representative of American Dentistry, with whom all will be satisfied and gratified.

He has the best wishes of all who knew him in this country, for his success and happiness.

---

All who were in active dental practice fifteen years ago, will remember Dr. A. Robertson, at that time of Wheeling.

He was then a very active member of the profession, and did much for its development and progress. He wrote one of the best treatises on extracting teeth that has ever been published.

During this period, time has brought serious changes to the Doctor; the curtain of darkness has fallen on him, not to be raised in this world; his material vision is gone and he rests in blindness. About eight years ago his eyes failed, and his other senses have to supplement those of the eyes so far as possible.

Through his liberality and want of desire and care for money, his adversity found him with very little preparation for the comforts and necessaries of life. The Doctor has not been disposed to complain of his condition, nor to assume the attitude of one begging for help; still it is a duty devolving upon the members of the profession, to which he was an honor, that all his wants are supplied, and that want never comes near enough to him to get a sight of his shadow.

It has been very rare indeed, that any such case has occurred in our profession, and the calls of duty upon our professional brethren, in any such direction have been few and far between; and we suggest that every one who has sympathy and love in his heart for his fellow in trouble, make some demonstration of that sympathy. Address him a note and let that enclose something that shall give emphasis to your words of kindness and cheer. The address is Dr. Abram Robertson, Georgetown, Mass.

